# Gut microbiome dysbiosis in men who have sex with men increases HIV infection risk through immunity homeostasis alteration

**DOI:** 10.3389/fcimb.2023.1260068

**Published:** 2023-11-16

**Authors:** Kangjie Li, Jielian Deng, Cong Zhang, Guichuan Lai, Biao Xie, Xiaoni Zhong

**Affiliations:** College of Public Health, Chongqing Medical University, Chongqing, China

**Keywords:** gut microbiome, men who have sex with men, HIV, gutMgene database, immunity

## Abstract

**Objectives:**

Recent studies pointed out that gut microbiome dysbiosis in HIV infection was possibly confounded in men who have sex with men (MSM), but there is a lack of evidence. It also remained unclear how MSM-associated gut microbiome dysbiosis affected human health. This study aimed to compare the differences in gut microbiome changes between HIV and MSM and reveal the potential impacts of MSM-associated gut microbiome dysbiosis on the immune system.

**Methods:**

We searched available studies based on the PubMed database, and all gut microbiome changes associated with HIV infection and MSM were extracted from the enrolled studies. The gutMgene database was used to identify the target genes and metabolites of the gut microbiome. Bioinformatic technology and single-cell RNA sequencing data analysis were utilized to explore the impacts of these gut microbiome changes on human immunity.

**Results:**

The results showed significant overlaps between the gut microbiome associated with HIV and that of MSM. Moreover, bioinformatic analysis revealed that gut microbiome dysbiosis in MSM had an impact on several pathways related to immunity, including the IL-17 signaling pathway and Th17 cell differentiation. Additionally, target genes of MSM-associated gut microbiome were found to be highly expressed in monocytes and lymphocytes, suggesting their potential regulatory role in immune cells. Furthermore, we found that MSM-associated gut microbiome could produce acetate and butyrate which were reported to increase the level of inflammatory factors.

**Conclusion:**

In conclusion, this study highlighted that MSM-associated gut microbiome dysbiosis might increase the risk of HIV acquisition by activating the immune system. Further studies are expected to elucidate the mechanism by which gut microbiome dysbiosis in MSM modulates HIV susceptibility.

## 1 Introduction

HIV infection has long been a global public health issue, with an increasing number of new infections in Eastern Europe and central Asia and the Middle East and North Africa, according to UNAIDS (The Path That Ends Aids, 2023). Men who have sex with men (MSM) were the key population that promoted HIV transmission in China (Dong et al., 2019). In the USA, MSM accounted for approximately 70% of HIV new infections in 2019, with the main transmission pathway being receptive anal intercourse (https://www.cdc.gov/). In addition to exploring behavioral aspects, understanding the biological aspects of the infection can provide valuable insights for the development of effective prevention and treatment strategies for HIV infection. The gut microbiome is established in the colon, and could inform human immune activity (Gury-BenAri et al., 2016), and the impact of gut microbiome dysbiosis on virus infection has aroused researchers’ interest (Fakharian et al., 2023). Recent research reported gut microbiome dysbiosis in patients with HIV infection (Lozupone et al., 2013; Dillon et al., 2014; Sortino et al., 2020). HIV-associated gut microbiome was closely related to activated T cells and increased inflammation (Mutlu et al., 2014) and could predict the immune status of HIV-infected patients (Nowak et al., 2015). Gut microbiome dysbiosis of HIV infection was characterized by decreased diversity, higher relative abundance of *Proteobacteria* phylum, and lower *Clostridium (* Yu et al., 2014). Nevertheless, the association between HIV infection and gut microbiome has been controversial due to various influencing factors, such as antiretroviral therapy, viral load, substance use, and hyperosmolar lubricant application (Pinto-Cardoso et al., 2017; Vesterbacka et al., 2017; Fulcher et al., 2018; Haaland et al., 2018; Cook et al., 2019a; Cook et al., 2019b). Specifically, because of the high proportion of MSM in HIV-infected patients, the results of HIV-associated gut microbiome dysbiosis were possibly confounded by sexual orientation (Noguera-Julian et al., 2016). Meta-analysis results indicated that both HIV and MSM changed gut microbiome and MSM-associated dysbiosis was distinct from HIV (Zhou et al., 2020). Further studies have consistently demonstrated the robust influence of sexual behavior on the composition of the gut microbiome, and both HIV infection and MSM status independently contributed to the regulation of the gut microbiome (Vujkovic-Cvijin et al., 2020; Gebrebrhan et al., 2021). However, it’s unclear how gut microbiome changes associated with HIV differed from that of MSM.

Gut microbiome dysbiosis in MSM has the potential to increase HIV infection risk (Chen et al., 2021). An experimental study observed that fecal bacterial communities of HIV-negative MSM could activate human peripheral blood mononuclear cells (PBMC) and increase the level of inflammatory factors *in vitro* compared to HIV-negative men who have sex with women (MSW) (Neff et al., 2018). The proportion of blood CD104^+^ CD4^+^ T cells was more abundant in HIV-negative MSM than that of MSW and the expression of CCR5 on lamina propria T cells of the colon was also higher in HIV-negative MSM (Coleman et al., 2020), suggesting that MSM-associated gut microbiome could induce the influx of HIV-targeted T cells to colon tissues. Moreover, *Holdemanella*, one MSM-associated microbiome, could elevate the frequency of CCR5^+^ CD4^+^ T cells (Yamada et al., 2021). Furthermore, fecal bacterial communities from MSM also drove immune activation in mice and promoted the HIV infection frequency of lamina propria cells *in vitro (* Li S. X. et al., 2019). Cumulatively this evidence sheds light on the hypothesis that the MSM-associated gut microbiome might increase HIV infection risk through modulating immune function. However, the comprehensive understanding of the effects of MSM-associated gut microbiome on human immunity function remains obscure.

In the present study, we compared the differences in gut microbiome changes between HIV infection and MSM status by systematically searching and collecting gut microbiome dysbiosis data related to HIV infection and MSM status from previously published studies. To further investigate the effects of MSM-associated gut microbiome on human biological function, we utilized the gutMGene database to search for the target genes of MSM-associated microbiome. Bioinformatic analysis, such as KEGG enrichment analysis, was then used to explore the functions of these genes. Single-cell RNA sequencing analysis was conducted to elucidate the impact of MSM-associated gut microbiome on immune cells. Finally, we also extracted the metabolites of MSM-associated gut microbiome from the gutMGene database and investigated their roles through documented literature, aiming at exploring the potential mechanism of how these intestinal florae interacted with the host.

## 2 Materials and methods

### 2.1 Data source

Our gut microbiome data were extracted from previously published studies. To obtain these data, a systematic review of the published literature on gut microbiota dysbiosis related to HIV infection or MSM behavior was conducted. A comprehensive search was conducted in the PubMed database using relevant keywords, including the gut microbiome, HIV, MSM, men who have sex with men, and receptive anal intercourse (RAI), in combination with logical operators “AND” or “OR”. The inclusion criteria were as follows: 1) studies focused on these topics were included: gut microbiome changes compared patients living with HIV to controls living without HIV, gut microbiome changes compared MSM to heterosexual males, or gut microbiome changes compared men who had RAI behaviors to men never conducted RAI, 2) methods for gut microbiome sequencing could be 16S rRNA gene amplicon sequencing or metagenome, 3) the sample type could be rectal swab, fecal or colon biopsies, 4) studies that reported patients with other sexually transmitted infections were also included. Studies focusing on gut microbiome dysbiosis of metabolic diseases or fat issues in HIV patients were excluded. Reviews and meta-analyses were also out of consideration.

The sample type, sample size, HIV status, MSM status, and methods for sequencing of all enrolled articles were recorded. For the HIV-positive patients, information about their CD4 T cell counts and HIV viral load was also extracted. Gut microbiome changes regarding HIV, MSM, or RAI were extracted from all available information including results description, figures, and **supplementary tables**.

### 2.2 Identification of target genes and metabolites of gut microbiome

gutMGene is a database for target genes of gut microbes and microbial metabolites recording, developed by Harbin Medical University (Cheng et al., 2022). The target genes and metabolites of the gut microbiome were obtained from gutMGene v1.0 (http://bio-annotation.cn/gutmgene/). Firstly, we collected all gut microbiotas associated with MSM. These microbiotas were then uploaded to the gutMGene database searching for the microbiota’s target genes and metabolites respectively. Gut microbiotas that did not match any genes or metabolites were excluded in the subsequent analysis.

### 2.3 Gene Ontology and Kyoto Encyclopedia of genes and genomes enrichment analysis

Gene ontology (GO) and Kyoto encyclopedia of genes and genomes (KEGG) enrichment analysis were performed to analyze the function of target genes of gut microbiotas. The R package “clusterprofiler” was used to conduct the GO and KEGG enrichment analysis. GO terms and KEGG pathways with p < 0.05 were defined as the significant enriched terms and pathways respectively.

### 2.4 Protein-protein interaction network analysis

Protein-protein interaction (PPI) network analysis was performed through a “string” database to explore the possible interaction among genes. All available target genes were inputted into the string website (https://string-db.org/), and nodes without any connection were removed from the network. Cytoscape_v3.9.0 software was used to visualize the network.

### 2.5 Construction of gut microbiome-gene-metabolite network

To visualize the interactions among MSM-associated gut microbiotas, target genes, and metabolites, we further developed a gut microbiome-gene-metabolite-network. Thus, the network was built based on previously published associations rather than unified quantitative data, aiming to compile the information visually. Briefly, MSM-associated gut microbiotas were extracted from previously published studies, and the gutMGene database was searched for target genes and metabolites of these gut microbiotas. Network construction was implemented through the R package “igraph”. Nodes represented the gut microbiome and their target genes or metabolites. Edges were defined as the associations between gut microbiota and genes or metabolites, which were recorded in the gutMGene database. The node color represented the type of node, and the edge color depicted the alteration of genes.

### 2.6 Single-cell RNA sequencing data analysis

scRNA-seq analysis was conducted to explore the potential impact of MSM-associated gut microbiotas on immune cells. Single-cell RNA sequencing data of healthy donors’ peripheral blood mononuclear cells were obtained from the GEO database (dataset: GSE188172). To avoid potential bias of gender, we enrolled only single-cell RNA sequencing data from males in this dataset. The R package “Seurat” was used to process single-cell data. Cells with fewer than 600 features and more than 20 percent expression of mitochondrial genes were excluded. The data then was normalized. The reduction was conducted by PCA and tSNE. Variable features were identified by the *FindVariableFeatures* function with default parameters. Marker genes from published studies were used to annotate cell clusters.

## 3 Results

### 3.1 Characteristics of included studies

**Figure 1:**
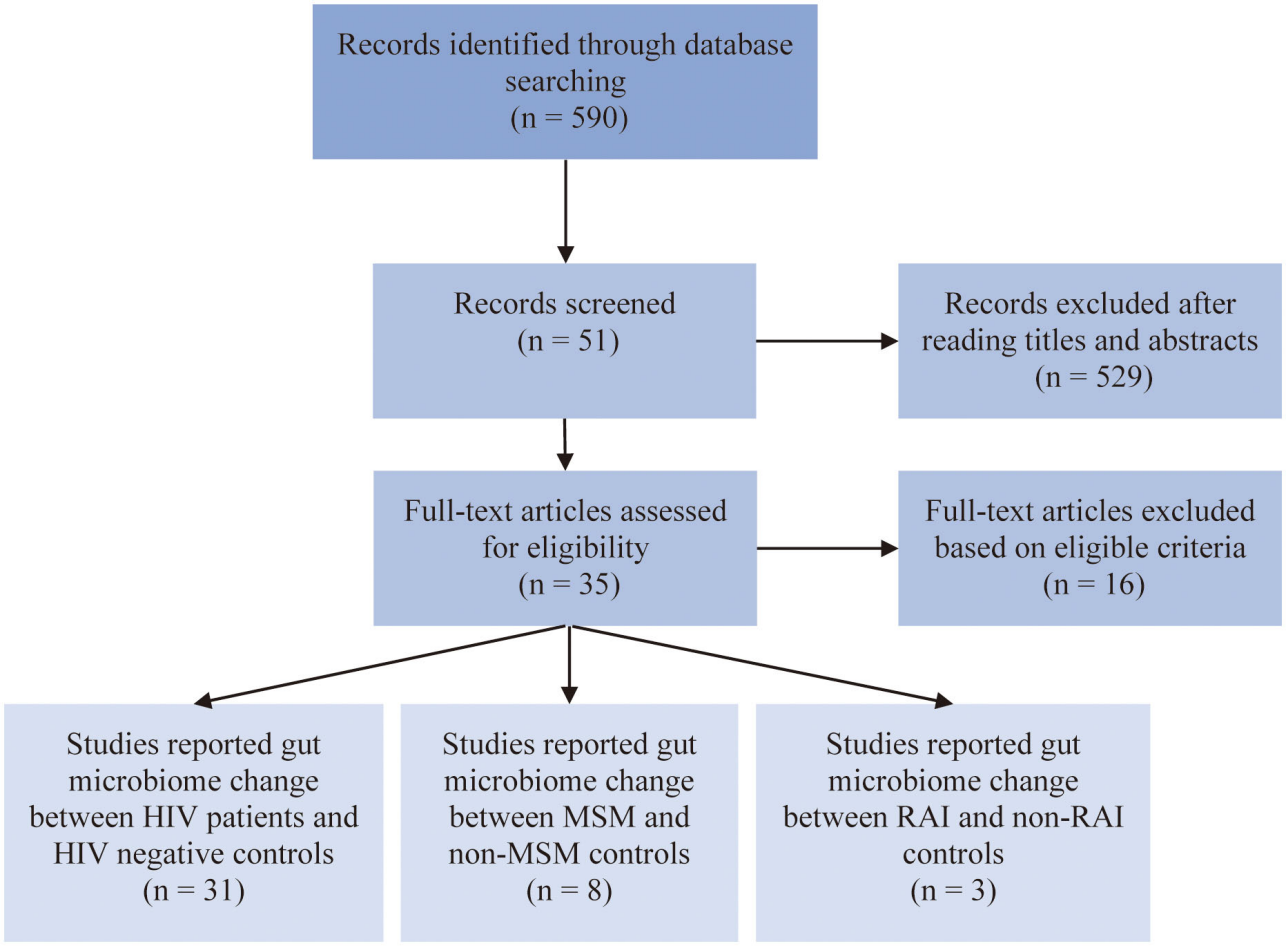
Flow chart of literature screening.

After carefully evaluation of related studies according to our inclusion criteria, 35 studies were enrolled in this analysis. The flow diagram of literature enrollment is depicted in **Figure 1**. Among these, 31 studies reported HIV-associated gut microbiome dysbiosis, 8 studies reported MSM-associated gut microbiome dysbiosis and only 3 studies focused on RAI-associated gut microbiome. The population in most of the studies was recruited from Europe and the USA, only a small percentage of the population were from Asia and Africa. Fecal samples were collected to detect gut microbiome through 16S rRNA gene sequencing by most studies. More detailed information on these studies can be found in **Table S1**.

### 3.2 HIV-associated gut microbiome changes were confounded by MSM

**Figure 2:**
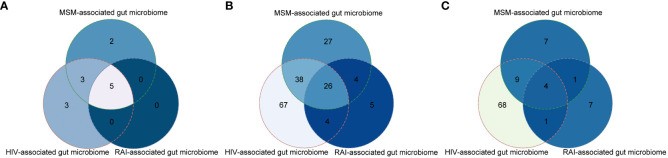
Venn plots showing large overlaps of gut microbiome changes among HIV infection, MSM, and RAI at the phylum level **(A)**, genus level **(B)**, and species level **(C)**.

To explore how HIV infection alters the gut microbiome and whether MSM confounds the former conclusion, we extracted HIV-associated gut microbiome and MSM-associated gut microbiome from all the available studies (**Table S2**). At the phylum level, 11 phyla were altered by HIV infection and *Firmicutes* were the most frequently altered phylum. The relative abundance of 10 phyla was significantly influenced by the MSM status. Like HIV infection, *Firmicutes* were the most frequently altered phylum by MSM. Eight phyla dysbiosis were shared by HIV infection and MSM, including *Firmicutes*, *Actinobacteria*, *Proteobacteria*, *Bacteroidetes*, *Euryarchaeota*, *Verrucomicrobia*, *Lentisphaerae*, and *Tenericutes* (**Figure 2A**). At the genus level, we found amount of genus taxa were altered by HIV infection, including *Bacteroides*, *Prevotella*, *Clostridium*, et al. Half of these genera, a total of 65, belonged to *Firmicutes* phylum. Eighteen genera belonged to *Bacteroidetes* phylum and 27 genera belonged to *Proteobacteria* phylum. While comparing the MSM group with the non-MSM group, 95 genera were associated with MSM, and most of these genera belonged to *Firmicutes* phylum. *Prevotella* was the most frequently reported genus in MSM-related gut microbiome dysbiosis, which was consistent with former research. Besides, the alteration of *Bacteroides*, *Collinsella*, *Blautia*, *Butyrivibrio*, *Holdemanella*, and *Clostridium* were also associated with MSM. Sixty-four genera were changed both in the HIV infection group and MSM group, including *Prevotella, Ruminococcus*, and *Holdemanella*. Our results showed that nearly half of the genus related to HIV infection were also altered by MSM, suggesting the confounding role of MSM (**Figure 2B**). We also collected gut microbiome change at the species level and 82 species and 21 species were found to be associated with HIV infection and MSM respectively. Thirteen species were both altered by HIV infection and MSM behavior (**Figure 2C**). However, due to the limitation of 16S rRNA gene sequencing, we didn’t find exact microbiome dysbiosis at the species level and the real situation might be underestimated.

We further hypothesized that gut microbiome dysbiosis in the MSM population probably resulted from RAI so we also extracted gut microbiome alteration associated with RAI as possible. The result showed most of the RAI-associated gut microbiome alterations were covered in the MSM-associated microbiome at the phylum, genus, and species level respectively (**Figure 2**), which supported our hypothesis. In summary, gut microbiome was altered by both HIV infection and MSM, but they were not the same pattern. Furthermore, recent HIV-associated gut microbiome dysbiosis was possibly confounded by MSM and more evidence is desired to study the association between gut microbiome homeostasis and sexual behavior.

### 3.3 MSM-associated gut microbiome were related to immunity activation

Except for gut microbiome dysbiosis itself, we were more interested in the potential influence of gut microbiome on human health. gutMGene database records numbers of target genes of gut microbiota and microbial metabolites from published research. Based on the gutMGene database, we searched for the target genes of MSM-associated gut microbiota, and then GO and KEGG enrichment analyses were conducted to investigate the impact of gut microbiome dysbiosis on human biological function.

**Figure 3:**
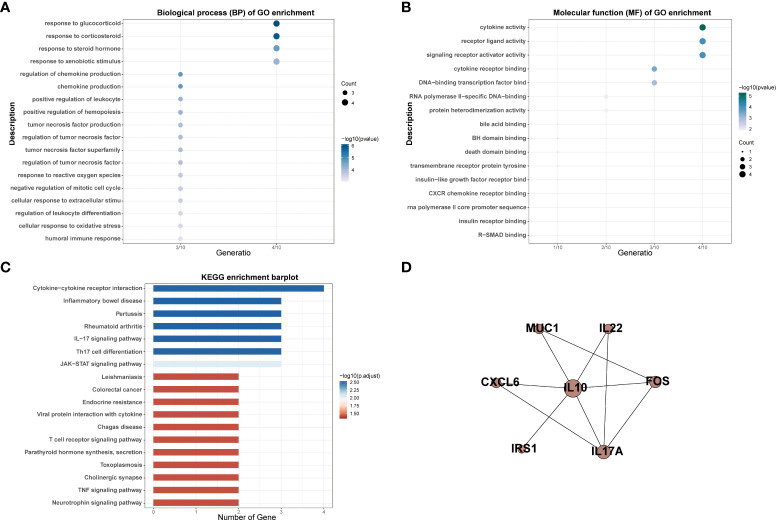
GO and KEGG enrichment analysis of targeted genes of MSM-associated gut microbiome **(A–C)**. PPI analysis indicated interactions among these genes **(D)**.

Thirteen MSM-associated gut microbiota were matched in the gutMGene database and 10 genes were targeted by these microbiotas, including BCL2, CXCL6, FOS, IL-10, IL17A, IL-22, IRS1, MUC1, NR1H4, and PLIN2 (**Table S3**). The results of GO analysis indicated that target genes of MSM-associated gut microbiota mainly enriched in cytokine activity, receptor-ligand activity, signaling receptor activator activity, response to glucocorticoid, response to corticosteroid, response to xenobiotic stimulus, and response to corticosteroid (**Figures 3A, B**). KEGG pathway analysis results showed these genes were enriched for variable immune pathways, including the IL-17 signaling pathway, Th17 cell differentiation, and JAK-STAT signaling pathway (**Figure 3C**). The above results demonstrated that MSM-associated gut microbiome dysbiosis influenced the expression of human genes and might arouse human immune activity.

We also performed PPI network analysis based on the string database to explore the interaction among targeted genes of MSM-associated gut microbiota. The results revealed significant interactions among these genes and IL-10 was the hub gene, which further hinted at the important role of MSM-associated gut microbiota in immunity (**Figure 3D**).

### 3.4 Target genes of MSM-associated gut microbiome were highly expressed in lymphocytes

**Figure 4:**
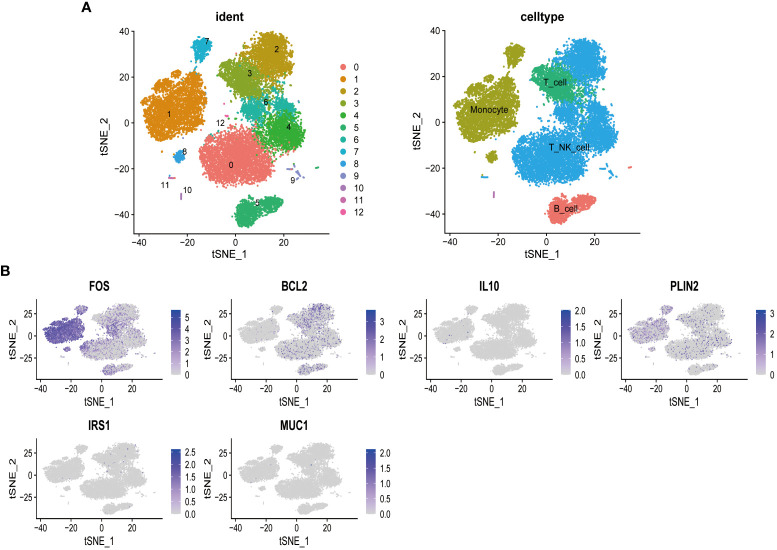
Single-cell sequencing analysis of peripheral blood mononuclear cells of healthy donors. T cells and natural killer cells were the most abundant cells **(A)**. Targeted genes of MSM-associated gut microbiome were highly expressed in lymphocytes and monocytes, indicating the regulation role of these gut microbiomes in immune activity **(B)**.

To further explore the influence of MSM-associated gut microbiome on the immune system, we performed scRNA-seq analysis, aiming at depicting the expression pattern of target genes of MSM-associated gut microbiome. After data preprocessing and reduction, thirteen clusters were identified and were annotated as B cell, T_NK_cell, and monocytes respectively (**Figure 4A**).

Among all target genes of MSM-associated gut microbiome, we found that FOS, a regulator of cell proliferation, differentiation, and transformation, was highly expressed among all clusters. BCL2 was specifically expressed in B cells and T_NK_cell, which indicated its roles in immune regulation. PLIN2 was relatively highly expressed in monocyte cells (**Figure 4B**). These results further demonstrated the important role of MSM-associated gut microbiome on immunity modulation.

### 3.5 MSM-associated gut microbiome might regulate immunity through altering metabolites

**Figure 5:**
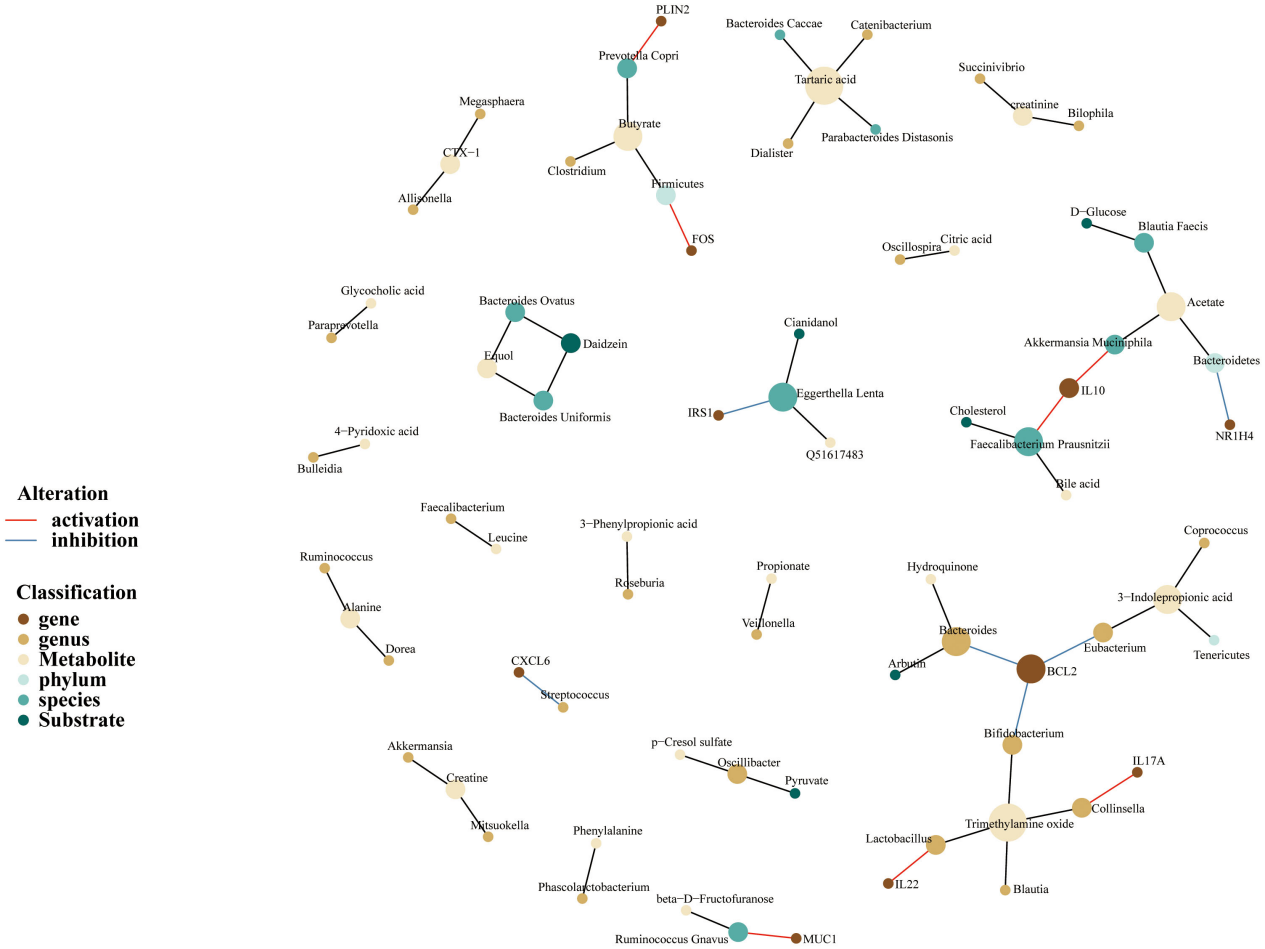
Gut microbiome-genes-metabolites network of MSM, depicting complex interactions among microbiome, genes, and metabolites. Nodes represent the gut microbiotas, genes or metabolites. Edges refer to the association between gut microbiotas and genes or metabolites. This network was built based on previously published associations rather than unified quantitative data, aiming to compile the information visually.

Because of the concept that gut microbiota takes part in regulating human biological function through its metabolites, we constructed a microbiome-gene-metabolites network to depict the relationship between gut microbiome and variable metabolites. As is shown in the following pictures, MSM-associated gut microbiome could metabolize a variety of substrates and produce corresponding metabolites (**Figure 5**). *Akkermansia Muciniphila* could metabolize acetate and upregulated the expression of IL-10. On the other hand, IL-10 was also regulated by *Faecalibacterium Prausnitzii* which metabolized cholesterol and produced bile acid. In the current network, BCL2, a hub node and an important targeted point for cancer therapy, was downregulated by 3 genera, including *Eubacterium*, *Bifidobacterium*, and *Bacteroides*.

In addition, the network depicted that acetate and trimethylamine oxide were the two important metabolites. We also noticed that *Prevotella Copri* species produced butyrate and enhanced the expression of PLIN2.

Overall, the above results demonstrated again that MSM-associated gut microbiome had the potential to regulate the human immune system. These microbiotas possibly work through metabolite alteration.

## 4 Discussion

In the present study, we found that gut microbiome dysbiosis existed both in HIV infection and MSM population, but with different characteristics. It’s worth noting that MSM-associated gut microbiome changes significantly overlapped with that of HIV, indicating the confounding role of MSM in gut microbiome changes associated with HIV infection. A recent well-designed study controlled various influencing factors, especially MSM status, and they found that HIV-associated microbiota was distinct from that of MSM (Vujkovic-Cvijin et al., 2020), which is consistent with our conclusion. However, due to the limitation of 16S rRNA gene sequencing, we did not observe enough gut microbiome changes at the species level in MSM and HIV cases. Moreover, our results prompted that MSM behavior could alter human immunity response through gut microbiome dysbiosis, this might be the potential mechanism of T-cell phenotype alteration and bioenergy metabolism disorder in the MSM population (Kruize et al., 2020). A recent study demonstrated that gut microbiome in MSM was associated with elevated inflammatory factors, which also supported our conclusion (Littlefield et al., 2022). Different from meta-analysis, our study systematically retrieved HIV and MSM-associated gut microbiome changes from available studies and focused on the influence of these gut microbiomes on human immunity by bioinformatic technology.

At the genus level, *Prevotella* was the most frequently reported changed microbiota both in HIV and MSM. *Prevotella* spp. and *Prevotella copri* were the most common species of *Prevotella* inhabiting the human intestinal tract. Although there is a lack of direct evidence, *Prevotella* was reported to be associated with inflammation, opportunity infections, and autoimmune diseases (Tett et al., 2021). *Prevotella* was also related to virus infection. Researchers found that the abundance of *Prevotella* was higher in women with high-risk HPV infection and associated with NF-KB signaling, suggesting its role in promoting virus infection by altering immune regulators (Dong et al., 2022). Future studies would be expected to explore the exact role of *Prevotella* on HIV infection and its mechanism.

In fact, we were more interested in the effects of gut microbiome dysbiosis on the host rather than the gut microbiome itself. In our analysis, multiple inflammatory factors were found to be targeted by MSM-associated gut microbiome, including IL-10, IL-17A, and IL-22. GO and KEGG enrichment analysis indicated immunity activation and aroused signaling pathway activity when gut microbiome dysbiosis occurred in MSM. Notably, target genes of MSM-associated gut microbiome were related to IL-17 signaling pathway and Th17 cell differentiation. IL-17 was an important pro-inflammatory cytokine secreted by T cells and was crucial for host defense, tissue repair, and inflammatory diseases (Li X. et al., 2019). IL-17 signaling activates inflammatory transcription factors to induce gene expression through the NF-KB pathway and MAPK pathway (Amatya et al., 2017). Besides, IL-17 could induce neutrophils through an indirect mechanism and was reported to link with viral immunity (Peng et al., 2017). For example, IL-17 signaling participated in the inflammatory response during SARS-CoV-2 infection (Wu et al., 2022). Low expression of IL-17 might be the key point of reduced susceptibility to HIV infection (Chege et al., 2012). This evidence suggested that MSM-associated gut microbiome had the potential to trigger inflammation to increase the risk of HIV acquirement. On the other hand, HIV preferentially depleted Th17 cells which produce IL-17 cytokines. Th17 cells produce more viral capsid proteins and lack RNases to promote HIV productivity (Christensen-Quick et al., 2016), further demonstrating the important role of gut microbiome dysbiosis of MSM in HIV infection risk.

To deepen the understanding of the effects of MSM-associated gut microbiome on the immune system, we further explored the expression pattern of target genes of MSM-associated gut microbiome through scRNA sequencing data analysis. PLIN2 is a member of the perilipin family, working as a structural protein that surrounds lipids to form lipid droplets (LD) (Kaushik and Cuervo, 2015), and PLIN2 was associated with chaperone-mediated autophagy. The overexpression of PLIN2 protected LD against autophagy and macrophages were the main source of PLIN2 in tumor microenvironment, researches also reported that high expression of PLIN2 induced immune suppression (He et al., 2022). In our analysis, we found PLIN2, which was activated by *Prevotella copri*, was highly expressed in monocytes, indicating that *Prevotella copri* might restrain the phagocytic function of monocytes and inhibit HIV elimination to promote its invasion. Furthermore, in our results, BCL2 was mainly expressed in T cells, B cells, and NK cells of PBMC of healthy donors. This gene encodes an integral outer mitochondrial membrane protein to block the apoptotic death of lymphocytes (Warren et al., 2019). We found that BCL2 was inhibited by several MSM-associated microbiomes, which might impair the stability of lymphocytes. Overall, MSM-associated gut microbiome dysbiosis had a great impact on human immune cells and contributed to HIV infection.

The gut microbiome takes part in sustaining human biological homeostasis possibly through its metabolites. Here we also extended to discuss the potential mechanism of how MSM-associated gut microbiome affects immune stability. Butyrate is a kind of ketone body, and we found that MSM-associated *Prevotella copri* could produce this compound. Butyrate acts as an inhibitor of histone deacetylase (HADC) and was reported to promote the production of pro-inflammatory factors to maintain gut homeostases, such as IL-10 in Th1 cells and IL-22 in CD4^+^ T cells (Sun et al., 2018; Yang et al., 2020). Besides, butyrate could also constrain the function of neutrophils to decrease proinflammatory cytokines in inflammatory bowel disease patients (Li et al., 2021). The above evidence prompted that MSM-associated gut microbiome regulated a variety of immune cells by producing metabolites, and the fluctuation of these metabolites undoubtedly disturbed the normal function of immune cells.

There were however limitations in our study. Firstly, gut microbiome data originated from 16S rRNA gene sequencing in most of our enrolled studies, which limited our understanding of MSM-associated gut microbiome at the species level. We encourage future studies to explore the association between the gut microbiome and MSM behavior by shotgun metagenomic sequencing. Secondly, the target genes of MSM-associated gut microbiotas were obtained from public databases, and the target genes of some microbiotas were not recorded. Thus, the impacts of MSM-associated gut microbiotas on human immunity were not fully understood. More evidence is expected to reveal how MSM-associated gut microbiome dysbiosis affects the immune system. Specifically, multi-omics study and bioinformatic technology may advance our understanding of this field. Thirdly, most of our enrolled studies did not report whether MSM participants received pre-exposure prophylaxis (PrEP) treatment, and the sample size of these studies was also relatively small. Thus, due to the limited sample size and other potential bias factors in our enrolled studies, the intestinal microbiota of the MSM population needs further research.

To sum up, our study systematically compared the differences in gut microbiome changes between HIV infection and MSM. We pointed out that HIV-associated gut microbiome change was confounded by MSM and more well-designed studies were expected to explore the exact gut microbiome dysbiosis in MSM. We also concluded that MSM-associated gut microbiome changes activated immune reactions to increase the risk of HIV infection, through regulating the level of their metabolites in the human body. Studies are desired to explore the mechanism of how MSM-associated gut microbiome increases the risk of HIV acquirement. We believe this work could help HIV therapy and prophylaxis, accelerating the achievement of the HIV elimination goal of World Health Organization.

## Data availability statement

Publicly available datasets were analyzed in this study. The dataset can be found in the GEO database using the following ID: GSE188172.

## Author contributions

KL: Data curation, Formal analysis, Writing – original draft, Writing – review & editing. JD: Data curation, Formal analysis, Writing – original draft, Writing – review & editing. CZ: Data curation, Writing – review & editing. GL: Data curation, Writing – review & editing. BX: Conceptualization, Funding acquisition, Writing – review & editing. XZ: Conceptualization, Supervision, Writing – review & editing.
